# Human 3D Cultures as Models for Evaluating Magnetic Nanoparticle CNS Cytotoxicity after Short- and Repeated Long-Term Exposure

**DOI:** 10.3390/ijms19071993

**Published:** 2018-07-08

**Authors:** Uliana De Simone, Marianna Roccio, Laura Gribaldo, Arsenio Spinillo, Francesca Caloni, Teresa Coccini

**Affiliations:** 1 Laboratory of Clinical and Experimental Toxicology, Toxicology Unit, ICS Maugeri SpA-BC, IRCCS Pavia, 27100 Pavia, Italy; uliana.desimone@icsmaugeri.it; 2 Department of Obstetrics and Gynecology, IRCCS Foundation Policlinico San Matteo and University of Pavia, 27100 Pavia, Italy; M.Roccio@smatteo.pv.it (M.R.); spinillo@smatteo.pv.it (A.S.); 3 European Commission, Directorate General Joint Research Centre, Directorate F-Health, Consumers and Reference Materials, Chemicals Safety and Alternative Methods Unit, 21027 Ispra, Italy; Laura.GRIBALDO@ec.europa.eu; 4 Università degli Studi di Milano, Dipartimento di Medicina Veterinaria (DIMEVET), 20133 Milano, Italy; francesca.caloni@unimi.it

**Keywords:** Fe_3_O_4_NPs, in vitro screening, D384 cells, SH-SY5Y cells, neurotoxicity, nanotoxicology

## Abstract

Since nanoparticles (NPs) can translocate to the brain and impact the highly vulnerable central nervous system (CNS), novel in vitro tools for the assessment of NP-induced neurotoxicity are advocated. In this study, two types of CNS spheroids have been developed from human D384 astrocyte- and SH-SY5Y neuronal-like cells, and optimized in combination with standard assays (viability readout and cell morphology) to test neurotoxic effects caused by Fe_3_O_4_NPs, as NP-model, after short- (24–48 h; 1–100µg/ml) and long-term repeated exposure (30days; 0.1–25µg/ml). Short-term exposure of 3D-spheroids to Fe_3_O_4_NP induced cytotoxicity at 10 µg/mL in astrocytes and 25 µg/mL neurons. After long-term repeated dose regimen, spheroids showed concentration- and time-dependent cell mortality at 10 µg/mL for D384 and 0.5 µg/mL for SH-SY5Y, indicating a higher susceptibility of neurons than astrocytes. Both spheroid types displayed cell disaggregation after the first week of treatment at ≥0.1 µg/mL and becoming considerably evident at higher concentrations and over time. Recreating the 3D-spatial environment of the CNS allows cells to behave in vitro more closely to the in vivo situations, therefore providing a model that can be used as a stand-alone test or as a part of integrated testing strategies. These models could drive an improvement in the species-relevant predictivity of toxicity testing.

## 1. Introduction

Nanotechnology is a sector where aspects related to impact on human and animal health and on the environment are of emerging concern, together with the result of possible consumer exposure to many nanoproducts present on the market [1,2] by different routes such as inhalation, ingestion, and dermal contact. Adverse effects on respiratory and cardiovascular systems following nanoparticles (NPs) exposure seem to be the most frequently reported [3,4]. 

Furthermore, several studies have also looked at NP impact on the highly vulnerable nervous system [5,6]. Available evidence suggests incomplete effectiveness of the blood brain barrier protection of the brain against NP translocation [5,7]. 

Regardless of the route of exposure, NPs could reach the blood vessels and translocate to the brain [8,9]. The distribution of NPs in the bloodstream also raises a particular concern of NP transfer from placenta to the fetal CNS [7] with serious damage as a consequence of direct exposure to NPs in utero [10].

A recent review warrants recognition of an emerging need to combine nanotoxicology and neurology and calls for novel specific tools and investigation methods for this discipline [7]. 

Although standardized procedures for the evaluation of NPs toxicity have not yet been defined, as a future task, the integration of validated in vitro-studies into safety assessment strategies is claimed by several Institutions because of animal welfare issues and the fact that in vitro testing is less expensive, time-consuming and laborious. Moreover, the increasing number of newly developed NPs entering the production and use quickly, in combination with the growth in societal risk aversion, is forcing gradually but steadily the shift from the traditional in vivo toxicological approaches towards alternative testing strategies including in vitro assays [11].

Currently, the in vitro approach used in nanotoxicology, involve either physico-chemical characterization of NPs or in vitro models (cellular and acellular), useful to clarify the mechanism of actions of NPs [12]. 

An important open question on nanoparticle safety is linked to the development of novel in vitro tools specific for the assessment of NPs neurotoxicity. The majority of current in vitro investigations on NP toxicity are performed mainly in 2D cell cultures [13]. With the advent of three-dimensional (3D) cell culture models, in vitro studies are now mimicking better the mammalian tissues properties in many aspects [14]. 

Human and environmental exposure to magnetic iron oxide nanoparticles (IONPs) as magnetite (Fe_3_O_4_NPs), represent an emerging risk, in relation to the increasing application in the industry (e.g., audio speaker, position sensing, water purification from heavy metals), for their specific properties [15,16,17]. Information on IONPs possible side effects is still unknown, therefore, it is urgent to investigate their impact on human health and ecosystem.

On the other hand, Fe_3_O_4_NPs have been also proposed for biomedical applications such as magnetic resonance (MR) imaging [18], drug/gene delivery [19,20], magnetic hyperthermia [21], biological separation [22], tissue engineering [23], stem cell labeling/tracking [24], and for advanced in vitro and in vivo cancer theranostic applications [25]. Thus, ideally in this “formulation” type, it should not influence cellular functions itself. For all these purposes, the NPs should act only as a delivery platform, and therefore, the intended NPs should be tested for toxicity.

Experimental studies indicate that Fe_3_O_4_NPs can reach the CNS independently of the route of administration (e.g., inhalation, intravenous, intraperitoneal) causing adverse effects in CNS [14,26,27]. 

In vitro studies have also supported and mechanistically detailed the Fe_3_O_4_NPs-induced neurotoxic effects. The majority of these studies have been performed on different CNS cell types using 2D culture model of rodent or human astrocytes and neurons. Results showed an intracellular iron uptake [28,29] associated with cell viability decrease [29,30,31,32], oxidative stress generation [31] and apoptosis [30].

Few studies have been published applying 3D spheroid model testing the toxicity of drugs including loaded supermagnetic iron nanoparticles (SPION) in glioblastoma cells [33] or in colon carcinoma cell line [34] and indicating that unloaded SPION did not affect the spheroid size. 

The use of spheroids as a model for screening of neurotoxins is fairly novel. 3D spheroid models with spontaneous self-organization provide useful tools for neuroscience because they can recreate aspects of the spatial and mechanical environment of the tissue, allowing cells to interact. The 3D spheroid model can be used for neurotoxin/drug screening and, additionally, for detection of effects caused by long repeated exposure at low concentrations, a condition that appears frequently in vivo. An additional advantage of applying the 3D cell culture in high-throughput (HT)-friendly 96-well format is the multiple re-dosing of the tested compounds and the possibility of a real-time monitoring of the effects. 

In this study we developed and optimized two types of CNS cell spheroid models using astrocytes and neurons, combining them with standard assays (i.e., viability readout and cell morphology) to test potential neurotoxic effects caused by nanoparticle exposure, in particular using Fe_3_O_4_NP as a NP model. This combination could provide more physiologically insightful information when compared to the two-dimensional (2D) systems. Specifically, cytotoxic effects on two separate 3D spheroid cultures from human brain cell lines, namely D384 astrocytes and SH-SY5Y neurons, exposed to different concentrations of Fe_3_O_4_NPs were evaluated after short-term exposure (24 and 48 h) to a single-dose, and long-term (intermittent) repeated exposure (for 30 days).

## 2. Results

### 2.1. Monitoring of 3D Astrocyte and Neuron Spheroids over a 30 Day Culture Period

**Figure 1:**
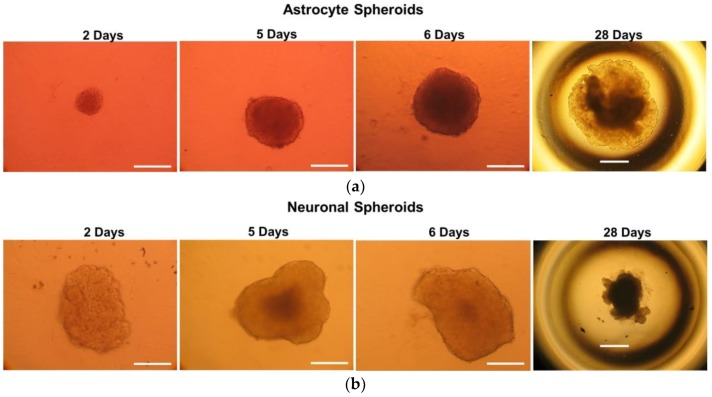
Microscopy images of astrocyte and neuronal spheroids: (**a**) Representative images of D384 spheroids by light field microscopy grown at the indicated time points. Scale bars: 100 μm for 2, 5 and 6 days; and 600 μm for 30 days, respectively; (**b**) Representative images of SH-SY5Y spheroids by light field microscopy grown at the indicated time points. Scale bars: 100 μm for 2, 5 and 6 days; and 600 μm for 30 days, respectively.

Spheroids were generated with D384 and SH-SY5Y cell lines. Figure 1a,b show light microscopic images of 3D spheroid astrocytes and neurons, respectively, cultured in ULA 96-well round-bottomed plates. 

D384 and SH-SY5Y cells, self-aggregated to form spheroid-like structures without the addition of exogenous extra-cellular matrix (ECM) components. Both brain cell types gradually formed well-defined spheroids centrally positioned in each well of the round bottom 96-well plates over a period of 5 and 6 days for SH-SY5Y and D384 cells, respectively. The shape of both cell type spheroids was largely unchanged up to 30 days while the size increased during time: diameter was about 200 and 1100 μm on day 6 and day 30, respectively, for D384 spheroids, and 300 and 800 μm on day 5 and 30, respectively, for SH-SY5Y spheroids (see also data below related to the growth monitoring during 30 days).

D384 cells formed spherical spheroids unlike SH-SY5Y cell line which formed spheroid phenotypes with elliptical or irregular shape. Moreover, D384 spheroids showed always a larger size than SH-SY5Y spheroids during the 30-day period. The reason of this faster growing could rely on a higher proliferation capacity of D384 compared to SH-SY5Y cells similarly to the behavior in 2D monolayer cultures in which the population doubling times are 9 and 48 h for D384 and SH-SY5Y cells, respectively. Based on the 2D proliferation rate characteristics, different initial seeding concentrations for the two types of cells were applied for the development of the specific type of 3D model.

### 2.2. Short-Term Exposure: Cytotoxicity Results after Single-Dose Treatment with Fe_3_O_4_NPs

#### 2.2.1. Cell Viability Evaluation by Trypan Blue Assay 

**Figure 2:**
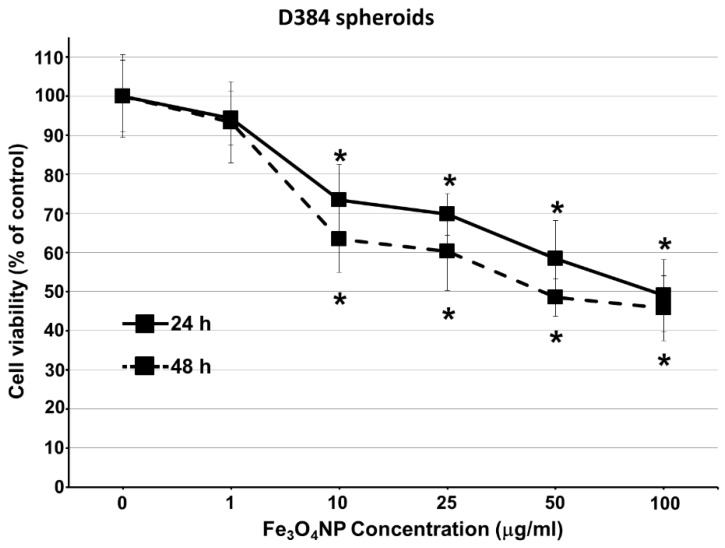
Cell viability evaluation in astrocyte spheroids after short-term exposure to Fe_3_O_4_NPs. Effect on cell viability evaluated by Trypan blue test in D384 spheroids exposed to different concentrations of Fe_3_O_4_NPs (1–100 μg/mL) after 24 and 48 h. Data are expressed as percentage of viable cells and represent the mean ± S.D. * *p* < 0.05, statistical analysis by two-way ANOVA followed by Dunnett’s test.

**Figure 3:**
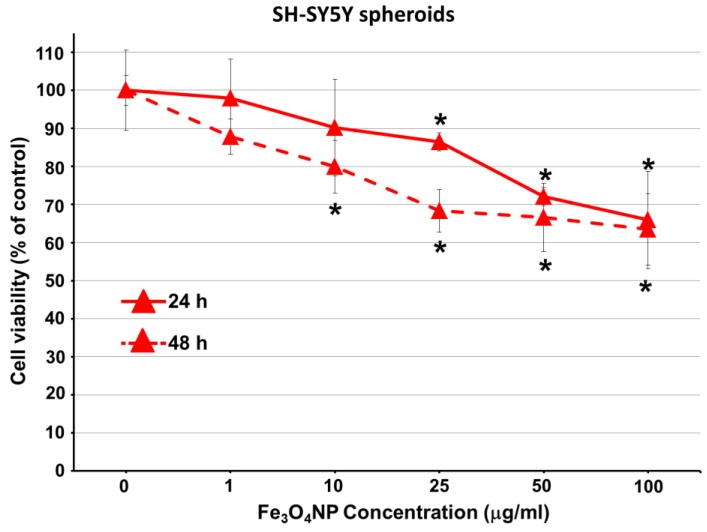
Cell viability evaluation in neuronal spheroids after short-term exposure to Fe_3_O_4_NPs. Effect on cell viability evaluated by Trypan blue test in SH-SY5Y spheroids exposed to different concentrations of Fe_3_O_4_NPs (1–100 μg/mL) after 24 and 48 h. Data are expressed as percentage of viable cells in treated cultures taking as 100% the number of viable cells in control condition and represent the mean ± S.D. * *p* < 0.05, statistical analysis by two-way ANOVA followed by Dunnett’s test.

Viability data of D384 and SH-SY5Y spheroids obtained using Trypan blue test after exposure to Fe_3_O_4_NP increasing concentrations are shown in Figure 2 and Figure 3. 

#### 2.2.2. Data on D384 Spheroids 

In D384 spheroids, Fe_3_O_4_NPs induced concentration-dependent cell mortality (Figure 2).

A significant decrease in cell viability (26%) was observed starting from 10 μg/mL after 24 h, and the maximum effect (about 50% mortality) was reached at the highest concentration (100 μg/mL). Following 48 h exposure, the cytotoxicity pattern was similar to that observed after 24 h, in particular Fe_3_O_4_NP concentrations ranging from 10 to 100 μg/mL were associated to a significant cell viability reduction of about 36–54%. Positive control (2.5 μM MeHg) induced significant reduction in cell viability (67.1% ± 5.0) after 48 h only.

#### 2.2.3. Data on SH-SY5Y Spheroids

SH-SY5Y spheroids were less susceptible to Fe_3_O_4_NPs than D384 spheroids: about 15–34% cell mortality was observed at the higher concentrations (25–100 μg/mL) after 24 h. Viability reduction was not further exacerbated after 48 h (20–37% reduction), although the effect started at the lower dose (10 μg/mL) (Figure 3). 

In SH-SY5Y spheroids, MeHg (2.5 μM) treatment induced a significant reduction of cell viability (35.0% ± 3.6) after 48 h only.

Preliminary studies have focused on 3D culture viability evaluation by standard colorimetric method based on tetrazolium reduction (MTT assay), traditionally used to assess number of viable cells in 2D cell culture. The findings indicated the not applicability of this assay: in acute exposure studies (after both 24 and 48 h), by MTT spectrophotometric evaluation, either after the standard 3h-DMSO or overnight-DMSO action, no effects were observed in both 3D SH-SY5H and D384 spheroids treated with Fe_3_O_4_NPs. These results are in accordance with other studies [35,36], underlining that in 3D spheroids tight cell-cell junctions can affect uptake and diffusion kinetics of a dye, therefore changing readout of the assay and making results more difficult to interpret [35,36].

#### 2.2.4. Morphological Analyses 

**Figure 4:**
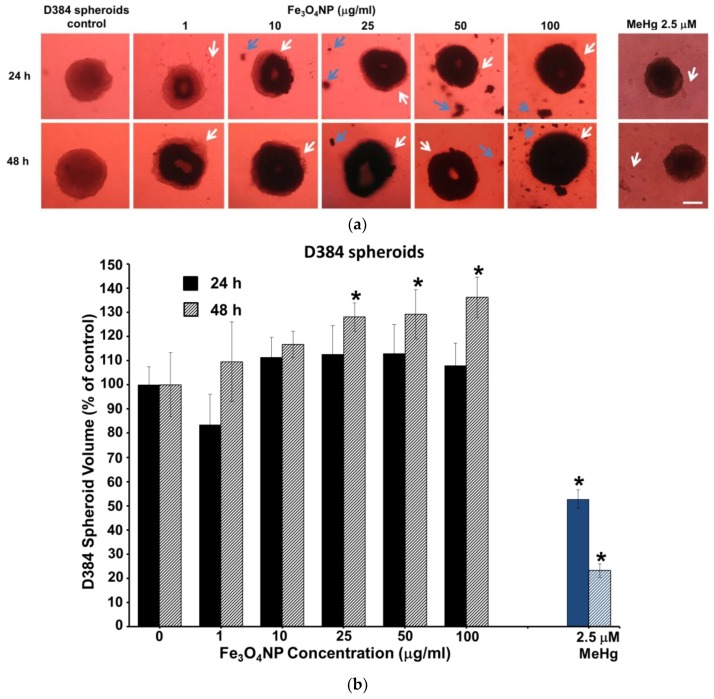
Morphology and volume of astrocyte spheroids after short-term treatment with Fe_3_O_4_NPs: (**a**) Bright-field images of D384 spheroids, washed with PBS, exposed to increasing concentrations of Fe_3_O_4_NPs after 24 and 48 h. White arrows indicate spheroid disaggregation and blue arrows indicate outside sediments of Fe_3_O_4_NPs. Scale bar: 100 μm; (**b**) Bar chart showing the volume growth (% of control) of D384 spheroids formed after 24 and 48 h, with error bars representing standard deviation of the mean (*n* = 4). * *p* < 0.05, statistical analysis by two-way ANOVA followed by Dunnett’s test.

**Figure 5:**
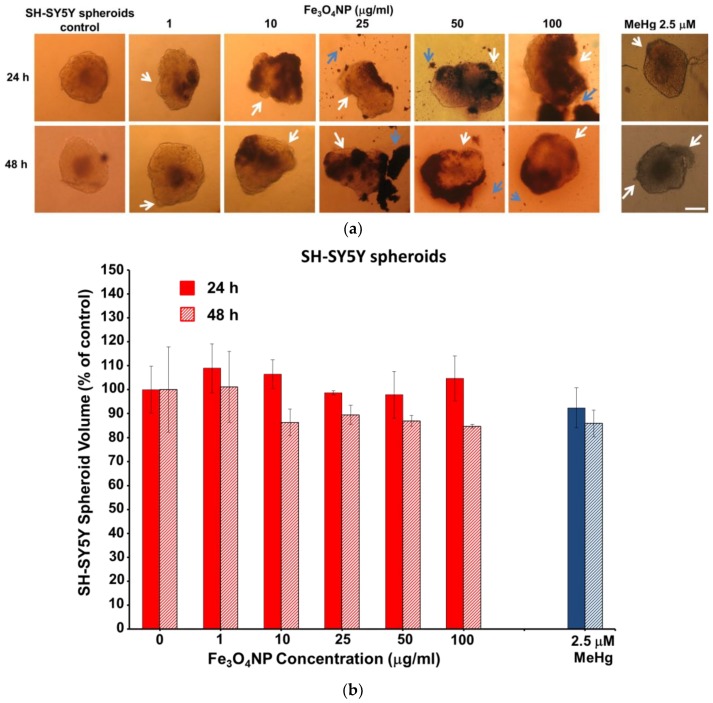
Morphology and volume of neuronal spheroids after short-term treatment with Fe_3_O_4_NPs: (**a**) Bright-field images of SH-SY5Y spheroids, washed with PBS, exposed to increasing concentrations of Fe_3_O_4_NPs after 24 and 48 h. White arrows indicate spheroid disaggregation and blue arrows indicate outside sediments of Fe_3_O_4_NPs. Scale bar: 100 μm; (**b**) Bar chart showing the volume growth (% of control) of SH-SY5Y spheroids formed after 24 and 48 h, with error bars representing standard deviation of the mean (*n* = 4).

Figure 4 and Figure 5 show representative images of randomly selected microscopic fields of both brain spheroids (D384 and SH-SY5Y cells) treated with increasing concentrations of Fe_3_O_4_NPs (1–100 μg/mL) for 24 and 48 h after the cleaning procedures in order to remove the excess NPs. 

In particular, light microscopy images of D384 spheroids indicated Fe_3_O_4_NPs accumulation in a concentration- and time-dependent manner; cells maintained sphere morphology although it was revealed a difference in cell packing density. Treatment resulted in a gradual disintegration of the spheroids, with cells detachment from the main body at all Fe_3_O_4_NPs concentrations and for each time point considered (Figure 4a).

Brownish sediments of Fe_3_O_4_NPs were also observed outside the spheroids.

Cell disaggregation was also evidenced as result of the toxic effect of the MeHg (2.5 μM) after 24 and 48 h exposure (Figure 4a).

Quantitative analysis by the assessment of the spheroid volumes indicated a concentration-dependent increase of the volume after 48 h: the increase started at 25 μg/mL (+25–40%) probably due to cell disaggregation around the spherical compact spheroid structure (Figure 4b). 

In SH-SY5Y spheroids, concentration- and time-dependent accumulation of Fe_3_O_4_NPs was also observed although in much less manner than in D384 spheroids, once again, associated with brownish sediments observable outside of spheroids and cell disaggregation (Figure 5a).

Apparently, the spheroid volumes did not change at any applied concentrations and both exposure times (Figure 5b).

Reduction of aggregate compactness of SH-SY5Y spheroids was also detected after MeHg (2.5 μM) treatment at both time point considered (24 and 48 h).

### 2.3. Long-Term Exposure: Cytotoxicity Results after Repeated Treatment with Fe_3_O_4_NPs for 30 Days

#### Cell Viability Evaluation by Trypan Blue Assay

**Figure 6:**
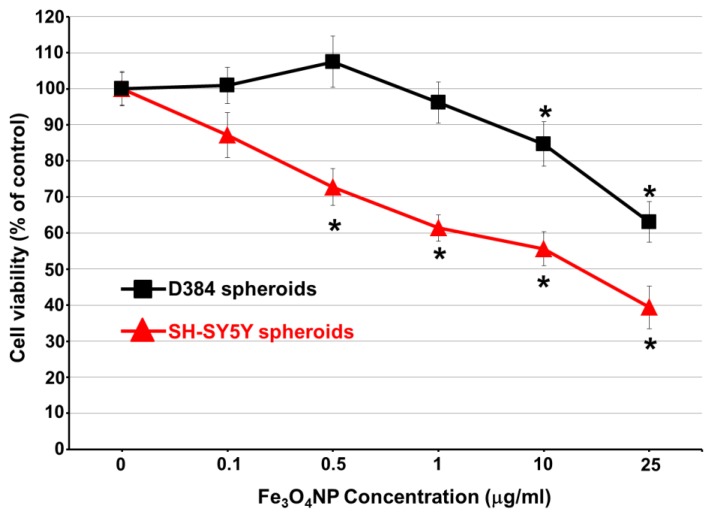
Cell viability evaluation in astrocyte and neuronal spheroids after long-term exposure to Fe_3_O_4_NPs. Dose-response curves of cell viability evaluated by trypan blue test. Astrocyte and neuronal spheroids were repeatedly treated with various low concentrations of Fe_3_O_4_NPs (0.1, 0.5, 1, 10, 25 μg/mL). Data are expressed as percentage of viable cells in treated cultures taking as 100% the number of viable cells in control condition and represent the mean ± S.D. * *p* < 0.05, statistical analysis by two-way ANOVA followed by Dunnett’s test.

In both D384 and SH-SY5Y spheroids, repeated exposure to low concentrations of Fe_3_O_4_NPs (0.1–25 μg/mL) caused a significant decrease of cell viability after 30 days (Figure 6): cell death started at the concentrations of 10 and 0.5 μg/mL, for D384 and SH-SY5Y spheroids. The latter, were more sensitive to Fe_3_O_4_NPs exposure compared to D384 spheroids: 25–60% cell viability decrease was observed in SH-SY5Y at Fe_3_O_4_NP concentrations ranging from 0.5 to 25 μg/mL (corresponding to cumulative total exposures from 6 to 300 μg/mL, respectively), while about 15–35% decrease of cell viability was observed in D384 spheroids starting at the higher concentrations of 10 and 25 μg/mL (corresponding to cumulative total exposures of 120 and 300 μg/mL, respectively).

When D384 and SH-SY5Y spheroids were repeatedly exposed to MeHg (1 μM), a significant decrease of cell viability (59.4 ± 4.2 and 65.5 ± 4.9% decrease in D384 and SH-SY5Y spheroids, respectively) was observed.

**Figure 7:**
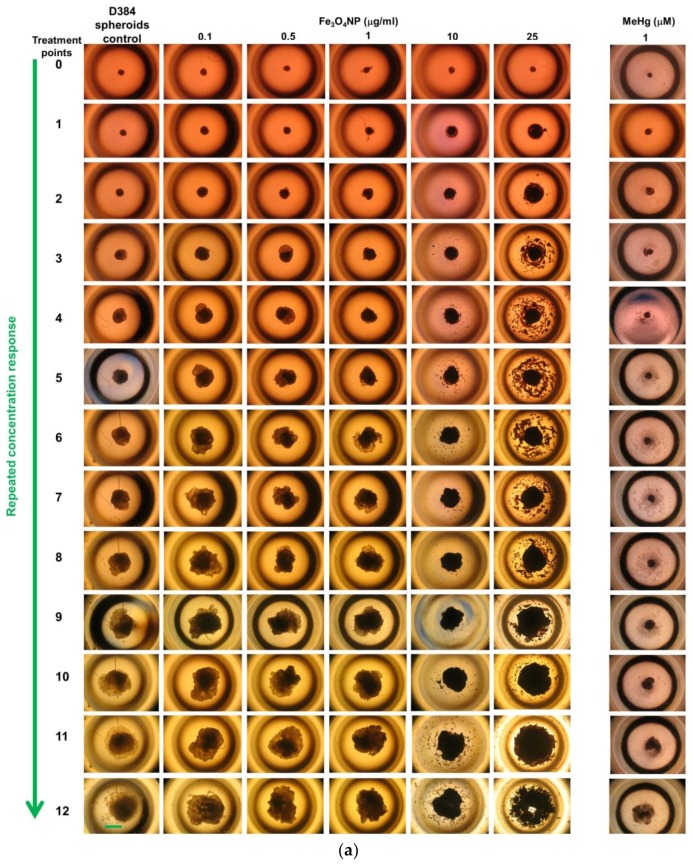

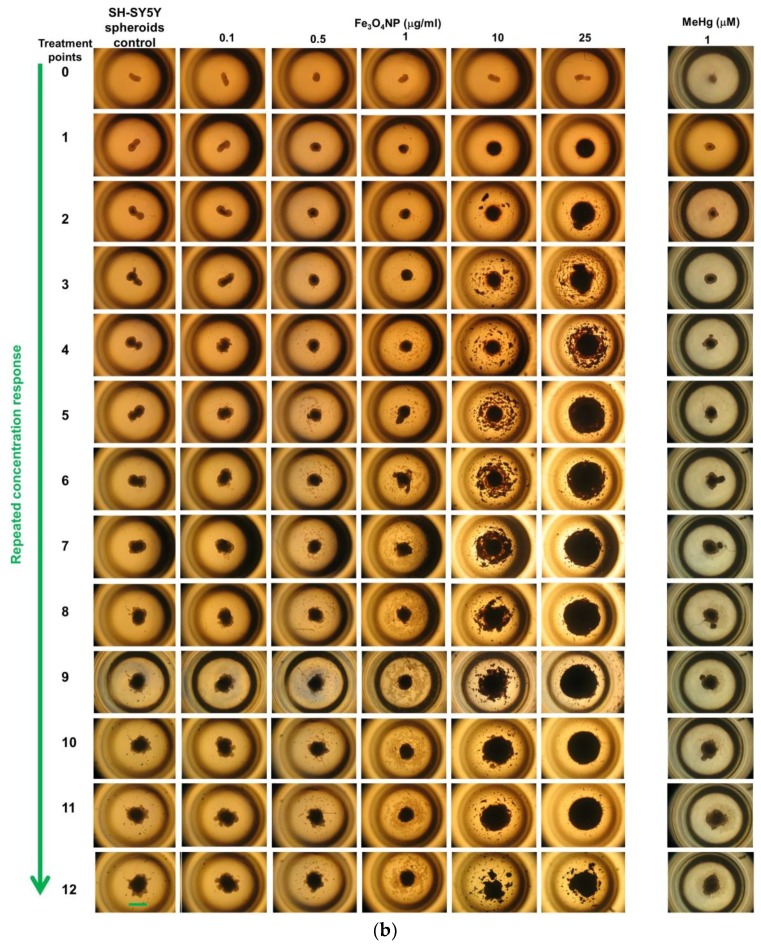
Morphology images in astrocyte and neuronal spheroids after long-term exposure to Fe_3_O_4_NPs: (**a**) Representative images of randomly selected microscopic light-fields of D384 spheroids over time (up to 30 days) with repeated treatment: twelve treatment points, at fixed low Fe_3_O_4_NPs concentrations (0.1; 0.5; 1; 10; 25 μg/mL), for a fixed period of exposure time (30 total days). Cell disaggregation was observed after the first week (i.e., meaning *n* = 3 repeated fixed concentration) starting from 0.1 μg/mL and became considerably evident at higher concentrations and over time (up to 30 days, corresponding to cumulative total exposures from 1.2 to 300 μg/mL). Scale bar: 600 μm; (**b**) Representative images of randomly selected microscopic light-fields of SH-SY5Y spheroids over time (up to 30 days) with repeated treatment: twelve treatment points, at fixed low Fe_3_O_4_NPs concentrations (0.1; 0.5; 1; 10; 25 μg/mL), for a fixed period of exposure time (30 total days). SH-SY5Y spheroids exhibited strong morphological alterations and lost the compactness of the structure already at the first week starting from 0.1 μg/mL and became evident at higher concentrations and over time (up to 30 days, corresponding to cumulative total exposures from 1.2 to 300 μg/mL). Scale bar: 600 μm.

Figure 7a,b showed representative images of randomly selected microscopic fields of both brain spheroids (D384 and SH-SY5Y cells) over time (up to 30 days) with repeated treatment: twelve treatment points, at fixed low Fe_3_O_4_NPs concentrations (0.1; 0.5; 1; 10; 25 μg/mL), for a fixed period of exposure time (30 total days) and corresponding to cumulative total exposures of 1.2; 6; 12; 120; 300 μg/mL.

D384 spheroids showed concentration-dependent accumulation of Fe_3_O_4_NPs: spheroids were increasingly loaded by Fe_3_O_4_NPs starting from the first week especially at higher concentrations (1–25 μg/mL) and become completely saturated by Fe_3_O_4_NPs over time (up to 30 days) as indicated by the brown-colored spheroids (Figure 7a). Fe_3_O_4_NPs induced similar effects in SH-SY5Y spheroids (Figure 7b).

Upon closer examination by over time monitoring, D384 spheroids failed to maintain their canonical shape and displayed cell disaggregation after the first week of repeated exposure (i.e., meaning *n* = 3 repeated fixed concentration) starting from 0.1 μg/mL (corresponding to cumulative total exposures of 1.2 μg/mL) and became considerably evident at the higher concentrations and over time (up to 30 days) (Figure 7a). Similarly, SH-SY5Y spheroids exhibited strong morphological alterations and lost the compactness of the structure: cell disaggregation was visible already at the first week starting from 0.1 μg/mL (corresponding to cumulative total exposures of 1.2 μg/mL) and became evident at the higher concentrations and over time (Figure 7b).

Both spheroids types (D384 and SH-SY5Y cells) after repeated exposure to 1 μM MeHg (corresponding to cumulative total exposures of 12 μM) grown smaller with decrease in cell packing density (i.e., cell disaggregation) in a concentration- and time-dependent manner (Figure 7a,b).

## 3. Discussion

3D in vitro models are becoming widely used when investigating drug/chemical toxicity. The present study demonstrated for the first time that 3D spheroids of astrocytes and neurons of human origin are useful for toxicity screening after short- and long-term repeated exposures to nanoparticles. 

Three-dimensional cell spheroids were generated using human D384 astrocytic and SH-SY5Y neuronal-like cells. In both cell lines spontaneous cell aggregation occurred using ULA 96-well round-bottomed plates. Additionally, conditions suitable to grow cell spheroids approximately 200–300 μm in diameter have been established. 

3D spheroids represent an innovative in vitro tool in toxicology, for their morphological and functional characteristics [37,38].

In this study, spheroids were treated with increasing concentrations of Fe_3_O_4_NPs. After short-term exposure (up to 48 h), both types of spheroids were affected by NP exposure: Fe_3_O_4_NPs induced a more pronounced concentration-dependent cell mortality in D384 spheroids (26–50% at concentrations ranging from 10 to 100 μg/mL), compared to that caused in SH-SY5Y spheroids (15–34% at the highest concentrations: 25–100 μg/mL). In accordance, higher concentration-dependent accumulation of Fe_3_O_4_NPs was observed in D384 than in SH-SY5Y spheroids. Brownish sediments were even visible outside both types of spheroid as well as the presence of disaggregated cells.

A more pronounced sensitivity of astrocytes in respect to neurons to Fe_3_O_4_NPs exposure have also been detected in a previous study assessing Fe_3_O_4_NP-induced toxicity in 2D mono-cultures (i.e., human SH-SY5Y neuroblastoma and D384 astrocytoma cells, by MTT assay and cell morphology) after short-term exposure (48 h) to different concentrations (1–100 μg/mL) [29] although the cytotoxicity was higher in each mono-cultured cell type than that observed in 3D spheroids: Fe_3_O_4_NPs induced 25–75% cell viability decrease in mono-cultured D384 astrocytes, and 35–45% cell mortality in SH-SY5Y neurons, in accordance with a more marked intracellular iron accumulation in astrocytes than in neurons. Recently, in mixed neural cell cultures, neurons have been shown to accumulate fewer IONPs than astrocytes [39].

Comparatively, when using 3D spheroid models, exhibiting features that are closer to the complex in vivo conditions, a significant attenuation of NP-induced toxicity was detected in both D384 and SH-SY5Y spheroids compared to that obtained in the respective mono-cultures. A similar trend in sensitivity was observed in a recent study carried out on human brain microvascular endothelial cells and showing different cytotoxic effects of superparamagnetic IONPs on cells grown in monolayer cell culture versus multicellular spheroids: viability was lesser in the 2D cell cultures compared to spheroids after exposure up to 72 h [40].

The differences between the two tested systems (2D versus 3D) may be partly due to the fact that cells grown as 3D spheroids, modeling the physiological 3D-architecture of tissues, possess a strongly deposited ECM, that provides cell-matrix interactions and the linking of secreted chemokines, growth factors, and a variety of signaling proteins in matrix-binding forms [38,41], and biochemical gradients which influence gradient-dependent cellular responses [42,41]. 

Moreover, 3D cultures compared to monolayers exhibit pronounced intracellular junctions mimicking physiological barriers [43], as well as the dense ECM with small pores, affecting the xenobiotic transport by decreasing their penetration [44,45]. Thus, as suggested, 2D systems may overestimate chemical toxicity, ascribable to the absence of a 3D organization, and confer some mechanical resistance to cytoskeletal disruption [46]. Several groups have begun to look at 3D spheroid culture systems for hepatocytes to assess the acute exposure to different drugs showing that 3D spheroids were clearly less sensitive than monolayer 2D cell cultures [47].

Importantly, one of the great advantage of using spheroids rely on the possibility to apply a repeated-dosing regimen, as we have confirmed their viability and functionality over a longer period (i.e., 30 days) compared to 2D monolayer cultures. The latter were given acute doses only, as these cells become over-confluent and lose viability once cultured for over 72 h, or 10 days maximum in the case of the clonogenic capacity evaluation. 

Groups that have started to apply 3D culture systems for the toxicity assessment in identifying hepatotoxic compounds have also applied this tool with repeated dosing regimens [48,49] providing the suitability of using this new in vitro high-throughput model for the prediction of hepatotoxicity. 

We also applied the repeated dosing schedule: in the long-term repeated exposure, astrocyte and neuronal spheroids showed concentration-dependent accumulation of Fe_3_O_4_NPs over time (up to 30 days) and cell mortality starting at the concentrations of 10 and 0.5 μg/mL, for D384 and SH-SY5Y spheroids, respectively. Neurons, in this case, showed a higher sensitivity to Fe_3_O_4_NPs compared to astrocytes.

By real-time monitoring, both spheroid types failed to maintain their canonical shape and displayed cell disaggregation starting within the first week of treatment at 0.1 μg/mL (corresponding to cumulative total exposures of 1.2 μg/mL) and became considerably evident at the higher concentrations and over the 30-day period. 

Altogether our findings indicated that both types of CNS cells, astrocytes, and neurons, are susceptible to Fe_3_O_4_NPs after both acute and long-term repeated dosing. In particular, a more pronounced susceptibility of astrocytic spheroids (similarly to the findings in 2D cultures acutely treated) has been observed after short-term exposure to Fe_3_O_4_NPs, whereas a higher susceptibility of neuronal spheroids was evidenced after prolonged and repeated exposure to low Fe_3_O_4_NP concentrations.

Fe_3_O_4_NPs may have produced iron liberation that exceeded the iron homeostasis capacity.

High level of iron in CNS, differently metabolized between neurons, astrocytes, oligodendrocytes, and microglia, can affect cell viability, as well as the transport and iron storage [50,51].

Astrocytes, considered key regulators of the iron metabolism in the brain, are able to rapidly accumulate iron ions and various iron-containing compounds, such as iron oxide NPs, by endocytotic mechanisms. Export of iron from astrocytes is also important for the supply of iron to other brain cell types, including neurons. Brain astrocytes deal well with an excess of iron explaining the reason why viability of astrocytes is less affected than neurons in a case of a prolonged insult. 

Iron plays a fundamental role in several neuronal functions in CNS including synaptic plasticity. Accordingly, neuronal iron supply is tightly controlled, and an excess of iron is detrimental for neuronal survival.

This in vitro study evidenced that the critical concentrations able to induce toxicity in spheroids (10 and 25 μg/mL for D384 and SH-SY5Y cells, respectively, after short-term exposure, and 10 and 0.5 μg/mL for D384 and SH-SY5Y cells, respectively, after long-term repeated dose regimen) are comparable to those measured in brain tissue of laboratory animals (rodents). In particular, Fe_3_O_4_NPs concentrations of 58 and 37 μg/g were detected in mouse brain, 3 and 10 days post-exposure, respectively, after a single intragastric administration (13 mg/mouse) [52]. Moreover, a cerebral regional distribution of Fe_3_O_4_NPs was also evidenced after intranasal instillation (20 μg for 7 days) in rats: Fe_3_O_4_NP concentrations deposited in the striatum and hippocampus areas were 0.050 and 0.040 μg/g, respectively, and more than half of these concentrations were retained at least 14 days post-instillation causing oxidative damage [31]. A recent in vivo study, also demonstrated that direct injection of the IONPs (10 μg/each brain hemisphere) into the dorsal striatum and hippocampus of mice, 7 and 14 days after surgery, decreased the tyrosine hydroxylase-positive fiber density in both the dorsal striatum and the hippocampus, and induced corresponding motor and memory deficits [53]. 

A possible correlation between neurodegenerative disorders and high iron level in brain has been suggested during aging as well as in other pathological conditions, although contribution of iron overload to pathology remains unclear [54,55]. In “Neurodegeneration with brain iron accumulation” (NBIA) disorders, which are often associated with severe dystonia and gait abnormalities, brain iron accumulation in the globus pallidus and other brain regions is reported. Alteration in iron metabolism is observed in aceruloplasminaemia and neuroferritinopathy, two diseases where mainly astrocytes and neurons were respectively involved [55].

The iron in excess in neurodegenerative tissues seems to be in the form of the magnetic Fe_3_O_4_ [56], hypothesis supported by the high concentration of Fe_3_O_4_ found in samples of Alzheimer’s disease tissue [57]. Recently, brain magnetite nanospheres were detected in human subjects and were consistent with an external, rather than an endogenous, source [58]. Their presence proves that externally sourced iron-bearing NPs can be transported directly into the brain, where they can pose a hazard to human health.

## 4. Materials and Methods 

### 4.1. Chemicals

Fetal bovine serum (FBS), culture medium and all cell culture reagents were purchased from Carlo Erba Reagents (Carlo Erba Reagents S.r.l., Cornaredo, Italy), 75 cm^2^ tissue culture flask and ULA 96-well round-bottomed plates were purchased from Corning (VWR International PBI, Milan, Italy). Trypan blue solution (0.4%) was purchased from VWR International PBI (Milan, Italy). Polyvinylpyrrolidonecoated Fe_3_O_4_NPs were obtained from nanoComposix (San Diego, CA, USA; lot no. MGM1837B). 

As a good laboratory practice, it was included, at each experimental session, an appropriate cellular process-specific control compound of known toxic effect, namely methylmercury (MeHg) at 1 and 2.5 μM (well-known to cause apoptotic and necrotic effects in CNS cells).

### 4.2. Fe_3_O_4_NP Suspension

Physico-chemical properties and morpho-dimensional analysis (by transmission electron microscopy) of polyvinylpyrrolidone-coated Fe_3_O_4_ nanoparticles stock suspension in 2 mM sodium citrate buffer were provided by the Company. Briefly, Fe_3_O_4_ stock suspension (20.6 mg/mL) was prepared by dissolving the powder in 2 mM sodium citrate solution. Fe_3_O_4_NPs showed roughly spherical, almost non-agglomerated particles, an average diameter of 19.07 ± 5.5 nm and a hydrodynamic diameter of 48.7 nm (by dynamic light scattering measurements). 

Fe_3_O_4_NP suspensions in the D384 culture medium (DMEM) and in SH-SY5Y culture medium (Ham’s F12) were as previously detailed [29]. Briefly, physico-chemical characterization of Fe_3_O_4_NPs in culture media (each specific for D384 and SH-SH5Y cells, respectively) was performed after 24 and 48 h at 10 and 25 μg/mL by dynamic light scattering. Fe_3_O_4_NPs were polydisperse (PdI ~ 0.8 (at 10 μg/mL) and ~0.7 (at 25 μg/mL) for both culture medium types) and not stable (zeta potential very close to zero for both culture medium types). At least three main subpopulations of particles were observed at 10 and 25 μg/mL for each culture medium type after 24 and 48 h: the first with a diameter about 10 nm, the second between 25 and 70 nm and the third about 700 nm.

Fe_3_O_4_NP treatment suspensions were prepared by diluting stock suspension (20.6 mg/mL) in culture media, then cells were exposed to target concentrations for short- and long-term repeated exposures. Fresh solutions of Fe_3_O_4_NPs were prepared and vortexed immediately before each use. 

### 4.3. CNS Cell Lines and Culture Conditions

All experiments were performed in standard cell culture conditions at 37 °C and 5% CO_2_.

Two tumor cell lines representative of CNS were selected: human astrocytoma cells (D384) and human neuroblastoma cells (SH-SY5Y). 

D384 cells were cultured in monolayer in media containing: DMEM supplemented with 10% (*v*/*v*) heat-inactivated fetal bovine serum (FBS), 2 mM L-glutamine, 50 IU/mL penicillin, 50 μg/mL streptomycin and 1% (*v*/*v*) sodium pyruvate (100 mM), and SH-SY5Y cells were cultured in monolayer in media containing: Eagle’s minimum essential medium and Ham’s F12 (1:1) with 15% heat-inactivated FBS, 2 mM L-glutamine, 50 IU/mL penicillin and 50 μg/mL streptomycin. Media were routinely changed three times weekly.

### 4.4. Spheroid Formation and Growth 

Among the different approach to enable 3D spheroid cultures, one is to use low-adhesion plates to promote the self-aggregation of cells into spheroids [59]. These plates not only have an ultralow attachment (ULA) surface coating to minimize cell adherence but also possess a well-defined geometry to drive and position a single spheroid within each well. Wells of low-adhesion plates have a round bottom with an ultralow cell attachment coating. The 96 ULA plates allow the natural disposition of cells to aggregate without the need for polymer scaffolds such as matrigel, polyglycolic acid, or microporous supports; they can also be used directly for the test treatment and evaluation. Experiments applying ULA plates for creating 3D spheroids allow testing of NPs in growth assays in a homogeneous size distribution, reproducibility, and a short formation time for the spheroids. 

3D spheroids cultures were created starting from single-cell suspensions obtained from trypsinized monolayers of both types of cells (D384 and SH-SY5Y) and diluted to the optimum cell plating density of 200 cells/200 μL for D384, and 10^4^ cells/200 μL for SH-SY5Y cells. Then, 200 μL of cell suspensions, of each type of cell line, were dispensed into each well of ULA 96-well round-bottomed plates. 

SH-SY5Y and D384 spheroids were cultured for 5 and 6 days, respectively, at 37 °C, 5% CO_2_, 95% humidity before Fe_3_O_4_NP treatment. Old media were carefully exchanged with fresh media on day 2 (50 μL/well) and 5 (100 μL/well) for SH-SY5Y and on day 6 (100 μL/well) for D384, taking care not to disturb the 3D spheroids. The initial optimal seeding densities (i.e., 200 cells/200 μL for D384, and 10^4^ cells/200 μL for SH-SY5Y cells) were established such that each spheroid for both cell lines (regardless of their proliferative potential and cell cycle time) fells within a size range of about 200 and 300 μm in diameter on day 6 and day 5 for D384 and SH-SY5Y, respectively. This size was chosen because it fits the requirements for gradients of oxygen, nutrients and proliferation rate that are essential for a biorelevant spheroid screen [60].

### 4.5. D384 and SH-SY5Y Spheroid Treatments and Cytotoxicity Evaluation (Trypan Blue Exclusions Test and Morphology)

At day 5 and 6, 3D spheroid cultures (SH-SY5Y and D384) were treated with different concentrations of Fe_3_O_4_NPs: ranging from 1 to 100 μg/mL for a single-dose exposure, and from 0.1 to 25 μg/mL for the repeated-dosing regimen. 

Cytotoxicity effects on both 3D spheroid-shaped neurons and astrocytes exposed to different concentrations of Fe_3_O_4_NPs were estimated evaluating 3D cell culture viability by trypan blue exclusion test, and spheroid morphology by microscopic evaluation after (i) short-term exposure (24 and 48 h) to a single dose and (ii) long-term exposure (for 30 days) to repeated fixed doses. The latter was performed by adding Fe_3_O_4_NPs (at fixed concentrations: 0.1, 0.5, 1, 10, and 25 μg/mL) three times per week, for 4 weeks, to 3D cultures (i.e., 100 μL medium/well was removed and replaced with Fe_3_O_4_NP treatment medium (100 μL/well) for a total of twelve doses (for each concentration) and carrying out the tests on day 30. This type of treatment allowed a real-time monitoring of the effects of Fe_3_O_4_NP treatment by continuously 3D cell morphology recording.

Control spheroids were treated with culture medium. 

### 4.6. Viability Assay by Trypan Blue Exclusion Test

Lacking a reference method specifically designed for 3D models, the Trypan blue exclusion test, is still one of the widely used cytotoxicity tests in this research field. In spheroids, at the end of each treatment (acute and prolonged exposure) with different Fe_3_O_4_NP concentrations, the culture media were carefully aspirated, and the spheroids washed with phosphate-buffered saline (PBS; 200 μL/well). Then each spheroid was disrupted using trypsin solution 1X (100 μL/well at 37 °C for up to 5 min) carefully pipetting up and down them, and 0.4% trypan blue solution was used in a ratio of 1:10 for the Trypan blue exclusion test. Ten μL of mixture were placed into Burker chamber and viable cells (cells do not take up trypan blue) counted manually in 4 main squares of the counting chamber delimited by three lines. Values of viability of treated cells were expressed as a percentage of that from corresponding control cells. The percentage of cell death was around 2–4% in untreated D384 and SH-SY5Y spheroids (controls) after 24 and 48 h, and around 10% after 30 days.

### 4.7. Growth Analyses of Spheroid Morphology by Light Microscopy

Both cell types (D384 and SH-SY5Y) in spheroids conditions were daily observed by light microscopy in order to monitor the size and growth of spheroids. Spheroids images were taken before Fe_3_O_4_NP treatments on day 2, 5 and 6 for both spheroid types (SH-SY5Y and D384). 

Brightfield images were then taken after 24 and 48 h exposure to single-concentration exposure to Fe_3_O_4_NPs (short-term treatment), and every other day after repeated-dose exposure to Fe_3_O_4_NPs.

Pictures were obtained by an inverted microscope (Zeiss Axiovert 25), using 10X objective (for short-term exposure) or 2.5X (for repeated long-term exposure) objectives, equipped with a digital camera (Canon powershot G8). Photographs were taken from each well and stored on the PC. 

The scale of images was determined using a calibration slide. Images and spheroid dimensions were performed as described by McMillan et al. [61]. Briefly, they were analyzed using the open-source software ImageJ by measuring the longest and shortest diameters (D1 and D2, respectively) for evaluation of the size. These values were then used to calculate the approximated volume (V) of each spheroid, estimated as V = 4/3 π × D1/D2 × (D2/2)^2.

### 4.8. Data Analyses

Data of the cytotoxicity effects are expressed as the mean ± SD of three separate experiments each carried out in four replicates for the acute exposure, and two separate experiments each carried out in 8 replicates for the chronic repeated exposure. 

Statistical analysis was performed by two-way ANOVA followed by Dunnett’s test. Only *p* values less than 0.05 were considered to be significant. 

## 5. Conclusions

These in vitro findings add value to the relevance of using new 3D in vitro cell-based models in toxicology and, specifically for our work, in the identification of the cytotoxicity of Fe_3_O_4_NPs. 

It is urgent to develop alternative methods for animal testing for safety assessment through an in vitro strategy based on screening tests as a predictive toxicological tool for nanomaterial evaluation.

Although in vivo validation remains the gold standard, an in vitro approach, supported by innovative 3D models, can accelerate the process by quickly identifying the most promising targets, establishing mechanisms of action, pathway of toxicity, and could be suitable to evaluate long-term exposure.

Recreating the 3D spatial environment of the CNS allows cells in vitro to behave more like their in vivo counterparts, providing robust and manageable model systems that mimic the cell biology present of the nervous system [62,63,64]. 

The use of spheroids as a model for investigating acute and chronic CNS injury induced by nanoparticles is fairly novel. In other areas of research (i.e., hepatotoxicity), spheroids are a well-established 3D cell culture technique. In our hands, SH-SY5Y and D384 cells formed reproducible well-rounded spheroids with uniform size, and functionality over a long period, showing their usefulness for assessing neurotoxicity induced by Fe_3_O_4_NPs.

These specific cellular 3D cultures may represent good models as “near-to-in vivo” and provide with a better and more realistic predictive value for safety and risk assessment of new and emerging materials (i.e., nanomaterials). 

Moreover, the 3D cell cultures are advantageous in that they not only enable drug safety and efficacy assessment in a more in vivo-like context than traditional 2D cell cultures, but also eliminate the species-specific differences (vs. animal models) that often impede interpretation of the preclinical effects in human systems.
